# Fine Particulate Matter Pollution and Hospital Admissions for Respiratory Diseases in Beijing, China

**DOI:** 10.3390/ijerph120911880

**Published:** 2015-09-22

**Authors:** Qiulin Xiong, Wenji Zhao, Zhaoning Gong, Wenhui Zhao, Tao Tang

**Affiliations:** 1 Urban Environmental Process and Digital Modeling Laboratory, Capital Normal University, Beijing 100048, China; E-Mails: xiong_ql@163.com (Q.X.); gongzhn@163.com (Z.G.); 2 Beijing Municipal Environmental Monitoring Center, Beijing 100044, China; E-Mail: wenhuidiandian@163.com; 3 Department of Geography and Planning, State University of New York College at Buffalo, Buffalo, NY 14222, USA; E-Mail: tangt@buffalostate.edu

**Keywords:** fine particulate matter, hospital admissions, respiratory diseases, functional areas, spatial distribution, grey correlation analysis

## Abstract

Fine particulate matter has become the premier air pollutant of Beijing in recent years, enormously impacting the environmental quality of the city and the health of the residents. Fine particles with aerodynamic diameters of 0~0.3 μm, 0.3~0.5 μm, and 0.5~1.0 μm, from the yeasr 2007 to 2012, were monitored, and the hospital data about respiratory diseases during the same period was gathered and calculated. Then the correlation between respiratory health and fine particles was studied by spatial analysis and grey correlation analysis. The results showed that the aerial fine particulate matter pollution was mainly distributed in the Zizhuyuan sub-district office. There was a certain association between respiratory health and fine particles. Outpatients with respiratory system disease in this study area were mostly located in the southeastern regions (Balizhuang sub-district office, Ganjiakou sub-district office, Wanshoulu sub-district office, and Yongdinglu sub-district office) and east-central regions (Zizhuyuan sub-district office and Shuangyushu sub-district office) of the study area. Correspondingly, PM_1_ (particulate matter with aerodynamic diameter smaller than 1.0 um) concentrations in these regions were higher than those in any other regions. Grey correlation analysis results showed that the correlation degree of the fine particle concentration with the number of outpatients is high, and the smaller fine particles had more obvious effects on respiratory system disease than larger particles.

## 1. Introduction

Though the proportion of fine particulate matter (PM_2.5_) in the global atmosphere is relatively low, it has a great influence on human health [1,2,3], as well as on the environment and climate. As the adsorptivity of PM_2.5_ is strong, they easily become the carrier of toxic substances in the air [4]. Those particles can cause respiratory diseases such as colds, asthma, upper respiratory infections, and pneumonia when they are inhaled into the lungs and remain in the blood. Inhalable particulate matter can affect the visibility of city, human health, and global climate during its transmission and transformation [5,6,7,8,9]. Hans *et al.* [10] evaluated the impact of PM_2.5_ on the morbidity and mortality of cardiovascular and respiratory diseases in Tallinn using fine spatial resolution and discrete model. Yin *et al.* [11] analyzed the concentration of inhalable particulate matter and numbers of outpatients with respiratory diseases in Shanghai during haze conditions. Ha *et al.* [12] analyzed the correlation between respiratory diseases and PM_2.5_ in Xining according to the numbers of outpatients with respiratory diseases and atmospheric pollution conditions in recent years. 

Air pollution, especially PM_2.5_ in Beijing, has received increasing attention in the past years. Despite the fact that Beijing has become one of the most atmosphere-polluted cities in the world, there has still been a lack of quantitative research regarding the health impact of PM_2.5_ in Beijing. Among the few scholars who have conducted PM_2.5_ health impact research, Tang *et al.* [13] diagnosed the exposure of the residential population and the vulnerable groups of children and elderly people to air particle pollution in urban Beijing. Liang *et al.* [14] investigated temporal patterns of PM_2.5_ over a five-year period and utilized the wavelet approach to explore the potential association between PM_2.5_ and influenza, on the basis of the collected data on hourly ambient PM_2.5_ from the years 2007 to 2012 and on monthly human influenza cases during the same period.

The reports about the influence of fine particulate matter on respiratory health mainly discuss the correlation from the viewpoint of epidemiology or toxicology [15,16,17,18,19,20,21,22,23]. There are few studies related to the correlation between fine particulate matter and respiratory health from the perspective of geography and spatial analysis, especially on the spatial relevance between PM_2.5_ and respiratory health in Beijing. Therefore, an urban area of Beijing was selected as the research area in the study. Additionally, the relationship between spatial distributions of PM_2.5_ and numbers of outpatients with respiratory diseases in Beijing from 2007 to 2012 was discussed, using the method of spatial analysis as well as grey correlation analysis. 

## 2. Data and Method

### 2.1. Study Area

**Figure 1:**
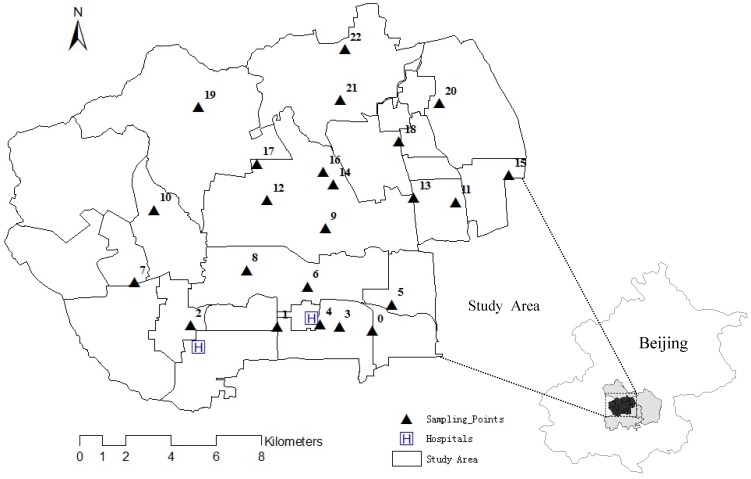
Study area and its location.

In recent years, the rapid economic and social development of Beijing has caught the attention of the world. However, Beijing's ecological environment, and the atmospheric environment in particular, has suffered a certain degree of pollution as a result of the limitation of natural conditions and human activities. For example, the geographic conditions that mountainous regions distribute in the west, north, and northeast work against the spread of atmospheric particulate matter. Moreover, the climate in Beijing is a typical temperate continental monsoon climate with temperature inversion happening frequently. Residents need heating in winter, so a huge amount of coal is burned during the heating period. Thus, it is easy to form atmospheric pollution in Beijing. Over the last 20 years, Beijing has faced a very serious atmospheric pollution problem due to energy consumption, the rapid growth in the number of vehicles, and the invasion of external pollution sources all through the year, especially in winter [24]. The study area is located in the center of Beijing (Figure 1). There are 24 sub-district offices, including 16 in the Haidian District and eight in the Shijingshan District (Table 1). Two large-scale general hospitals are located in the study area whose radiation scope covers all those sub-district offices. Thus, their outpatients’ information reflects the local residents’ health status. 

**Table 1:** Districts in the study area.

FID	ID	Name	District	Population	Post Code
0	87	Balizhuang	Haidian	128948	100036
1	88	Ganjiakou	Haidian	111596	100037
2	89	Wanshoulu	Haidian	148159	100036
3	90	Yongdinglu	Haidian	37394	100039
4	91	Yangfangdian	Haidian	151419	100038
5	100	Qinglongqiao	Haidian	145286	100091
6	101	Xueyuanlu	Haidian	287124	100083
7	102	Xiangshan	Haidian	35690	100093
8	103	Haidian	Haidian	143513	100080
9	104	Qinghuayuan	Haidian	49072	100083
10	105	Yanyuan	Haidian	36706	100080
11	106	Zizhuyuan	Haidian	222973	100044
12	107	Zhongguancun	Haidian	92900	100080
13	108	Shuangyushu	Haidian	50211	100089
14	109	Beitaipingzhuang	Haidian	136244	100088
15	110	Beixiaguan	Haidian	131720	100081
16	111	Bajiao	Shijingshan	70849	100049
17	112	Gucheng	Shijingshan	62249	100043
18	113	Laoshan	Shijingshan	37774	100049
19	114	Babaoshan	Shijingshan	100731	100040
20	115	Wulituo	Shijingshan	30664	100042
21	116	Pingguoyuan	Shijingshan	81852	100041
22	117	Guangning	Shijingshan	14365	100041
23	118	Jinding	Shijingshan	61963	100041

### 2.2. Data Description

Data of this study mainly include the following two parts: respiratory disease data and fine particulate matter data.

(1) Data of respiratory disease. Respiratory disease is a kind of familiar disease which exists mainly in the lungs, chest, trachea, and bronchus. The common respiratory diseases include acute upper respiratory infection, acute bronchitis, and so on. The daily respiratory outpatients’ information in this study was collected from two large-scale general hospitals which are located in the region (Figure 1). Twelve kinds of respiratory disease cases were collected, including upper respiratory infection (J06), obstructive pneumonia (J16), obstructive emphysema (J43), asthma (J45), chronic bronchitis (J42), chronic obstructive pulmonary disease (J44), chronic tonsillitis (J03), aspiration pneumonia (J03), acute bronchitis (J93), acute pharyngitis (J02), acute laryngitis (J04), acute purulent tonsillitis (J03), and respiratory failure (J96) [25]. The locations were spatialized by the information of addresses and zip codes provided by outpatients. The hospitals started to carry out the electronic medical records system from 2007 on, and data of outpatients with respiratory diseases was gathered from 2007 to 2012.

The administrative map scale of the study area is 1:25,000. The study area is divided into 24 units by sub-district office (Figure 1). Sub-district office was used as the basic unit while being analyzed. According to the sixth population census data in 2010, the population of the Xueyuanlu sub-district office in the Haidian District was the highest, reaching 287,124 (Table 1). Districts with high population density were distributed mainly in the west of the city where a majority of outpatients with respiratory diseases live. Thus, this research has a good representativeness. 

(2) Data of fine particulate matter. Handy Laser Particle Counter Kanomax MODEL 3886GEO-α was used to monitor the number concentrations of different sizes (0~0.3 μm, 0.3~0.5 μm, and 0.5~1.0 μm) of fine particles in the study area during 2007~2012. Simultaneously, the spatial coordinates of each sampling point were obtained using a handheld Trimble GPS. MODEL 3886GEO-α can have a 5-channel (0.3, 0.5, 1.0, 3.0, 5.0 μm) test at the same time, and the test report meets FS-209E, ISO14644-1, and other relevant environmental standards. Provided that the cascade is connected, this instrument will become a multi-point monitoring system. In this case, the sampling time, frequency, and interval can be set according to the need. When the particle number reaches 70 million per cubic meter, the consistency of loss is less than 5%. The sampling time was December to next January and June to July every year. During the sampling period of each year, the weather conditions were relatively stable, with less wind, no major storms blowing, and no heavy snow. In short, the weather system was relatively stable during sampling periods.

To make the data of the particulate matter representative, sampling points were arranged evenly, extensively, and regularly over different underlying surfaces in Beijing city, whose total number was 23, as shown in Figure 1. The sampling program followed the “Three Sames” principle, *i.e.* the same height (1.5 m above the ground), the same time (the sampling time was ensured to be the same), and the same distance (1.5 m away from the road). In the meantime, the handheld anemograph (Kestrel 3500) was used to record the temperature, relative humidity, and wind speed of the sampling points. All data acquired in the study area were imported into ArcGIS software in the form of shp (a kind of spatial data format).

### 2.3. Method

#### 2.3.1. Spatial Analysis

Spatial analysis is an important factor of geography and geographic information science which has received more and more attention in geography and its related disciplines [26]. It is the analysis technology that obtains the location, distribution, shape, formation, and evolution information of related geographic objects, as well as one of the core functions of the geographical information system. 

Firstly, the particle number concentration data derived from MODEL 3886GEO-α was associated with the spatial coordinates of the sampling sites measured by handheld GPS via the same attribute (object ID). Consequently, particulate matter data was assigned to spatial attributes. Secondly, geocoding analysis was conducted in ArcGIS software. Therefore, the spatial properties of the particulate matter data could form the spatial coordinates, whose projection was to coincide with the administrative demarcation diagrams of Beijing city. Finally, spatial analysis was carried out in the geographic information systems. After all those three steps were finished, the spatial distribution of the particle number concentration as well as its temporal evolution can be simulated. 

#### 2.3.2. Grey Correlation Analysis

To study the relationship between fine particulate matter and respiratory disease, the grey correlation method was utilized in this paper. Grey correlation analysis is used to quantize and analyze the development tendency of dynamic procedures by closeness of curve shape. The closer the geometry curve shape is, the greater the correlation of the comparison system is [27,28,29]. 

$S_{ij}=\left(x_{ij}^{0}\left(1\right),x_{ij}^{0}\left(2\right),\cdot \cdot \cdot ,x_{ij}^{0}\left(n\right)\right)$ is set as the reference sequence and $S_{i0}=\left(x_{i0}\left(1\right),x_{i0}\left(2\right),\cdot \cdot \cdot ,x_{i0}\left(n\right)\right)$ is the comparative sequence. The reference sequence reflects the characteristics of the system behavior and the comparative sequence is composed of factors that affect the system behavior. Usually, the initial values of the reference sequence and compared sequence have different units. Thus, it adopts the equalization method in dimensionless treatment before comparison. This is as follows.

(1) $$\bar{S_{ij}}=\frac{1}{m}\sum_{k=1}^{m}S_{ij}(k)$$

(2) $$S_{ij}(k)d=\frac{S_{ij}(k)}{\bar{S}}$$

In Equations (1) and (2), *k =* 0,1,…,*m*. Then the correlation coefficient of the reference sequence and compared sequence can be obtained.

(3) $$\zeta_{i}(k)=\frac{\min_{i}\;\min_{k}|S_{ij}(k)-S_{i0}(k)|+\rho \;\max_{i}\;\max_{k}|S_{ij}(k)-S_{i0}(k)|}{\left|S_{ij}(k)-S_{i0}(k)|+\rho \;\max_{i}\;\max_{k}|S_{ij}(k)-S_{i0}(k)\right|}$$

In Equation (3), *k* = 1,2,…,*n*. Thus, $\min_{i}\;\min_{k}|S_{i}\left(k\right)-S_{0}\left(k\right)|$ is the minimum value of the two stages and $\max_{i}\;\max_{k}|S_{ij}(k)-S_{i0}(k)|$ is the maximum value of the two stages; ρ is resolution ratio. Its value is between 0 and 1. Usually, ρ = 0.5.

Because the number of the correlation coefficient is large, in order to be convenient for comparing, the degree of correlation was calculated to judge the influence between the reference sequence and the compared sequence.

(4) $$\gamma =\frac{1}{m}\sum_{k=1}^{m}\xi_{i}(k)$$

In Equation (4), *k =* 0,1,…,*m*. 

## 3. Results and Discussion

### 3.1. Spatial Distribution of Fine Particulate Matter

**Figure 2:**
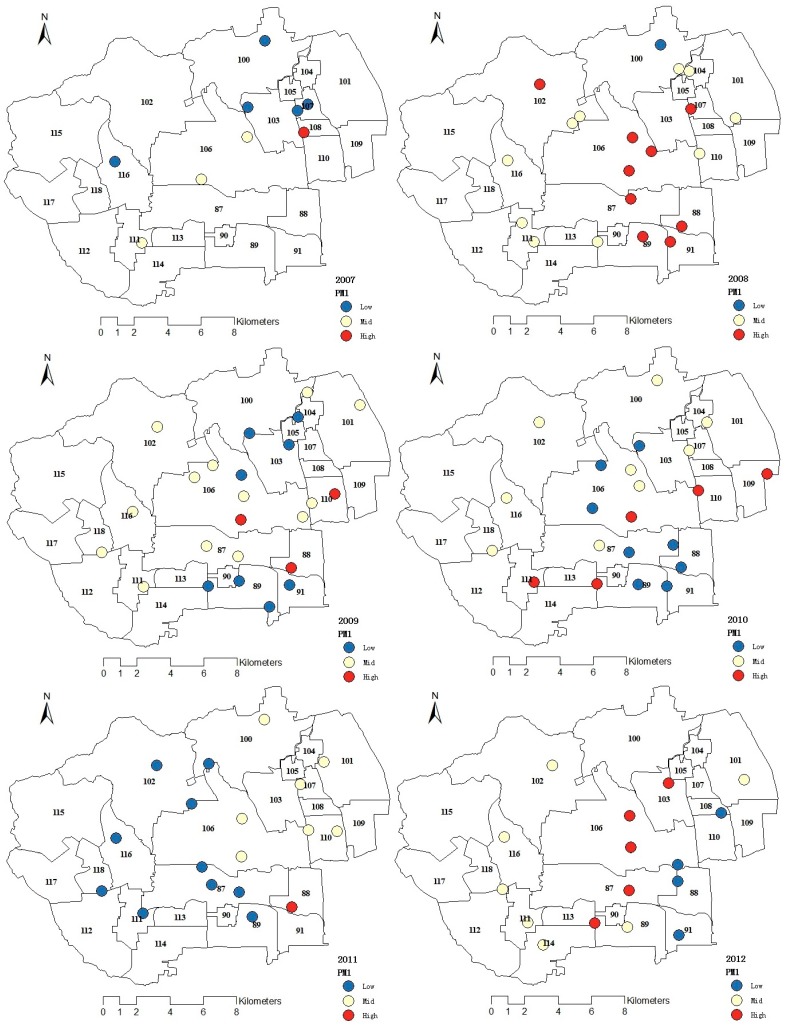
Spatial distribution of PM_1_ from 2007 to 2012.

The detailed information about the spatial distribution of PM_1_ from 2007 to 2012 was shown in Figure 2. In 2007, the most heavily PM_1_-polluting sub-district offices were Shuangyushu sub-district office and Zizhuyuan sub-district office. Correspondingly, the number of outpatients from these two sub-district offices were higher than those of other sub-district offices. In 2008, the more heavily PM_1_-polluting sub-district offices were Balizhuang sub-district office, Ganjiakou sub-district office, Wanshoulu sub-district office, and Zizhuyuan sub-district office. In 2009 and 2011, the more heavily PM_1_-polluting sub-district office was Zizhuyuan sub-district office. In 2012, the more heavily PM_1_-polluting sub-district offices were Babaoshan sub-district office and Zizhuyuan sub-district office. 

### 3.2. Respiratory Outpatients Distribution

Statistics on the numbers of outpatients in the respiratory department were taken from 2007 to 2012. The statistics variables are shown in Table 2, including Sum, Min, Max, Mean, Median, SD (standard deviation), and RSD (relative standard deviation) of the respiratory outpatients in the study area. According to Table 2, the largest number of the total outpatients (Sum) of all sub-district offices in a year appeared in 2010, reaching as high as 649; the smallest number was in 2007, with only 270. The minimum (Min) values of one single sub-district office were just 0 or 1. Particularly, there were four sub-district offices in 2008 where no respiratory cases took place. It was likely because the Beijing Olympic Games were held this year, and the ecological environment was better than in other years. The maximum (Max) values ranged from 82 (in 2008) to 153 (in 2011). The average (Mean) values ranged from 11 (in 2007) to 27 (in 2011). The median values ranged from 3 (in 2007) to 9 (in 2011). All these three variables showed an annually increasing trend. SD values ranged from 18.56 (in 2007) to 41.92 (in 2011). RSD was calculated by SD and Mean. All the RSD values were higher than 1.5 except those in 2009 (1.49), which demonstrated that the spatial distribution of outpatients in the study area was quite different during these years. The highest number of outpatients was 153 in the Wanshou Road sub-district office in 2011. This sub-district office is also a high population density area in Beijing.

**Table 2:** Statistics on respiratory outpatients from 2007 to 2012.

Statistics Year	2007	2008	2009	2010	2011	2012
Sum	270	316	582	649	639	623
Min	1	0	0	1	1	0
Max	82	82	131	147	153	127
Mean	11	13	24	27	27	25
Median	3	5	9	9	9	8
SD	18.56	22.80	36.10	41.41	41.92	38.12
RSD	1.65	1.73	1.49	1.53	1.57	1.54

**Figure 3:**
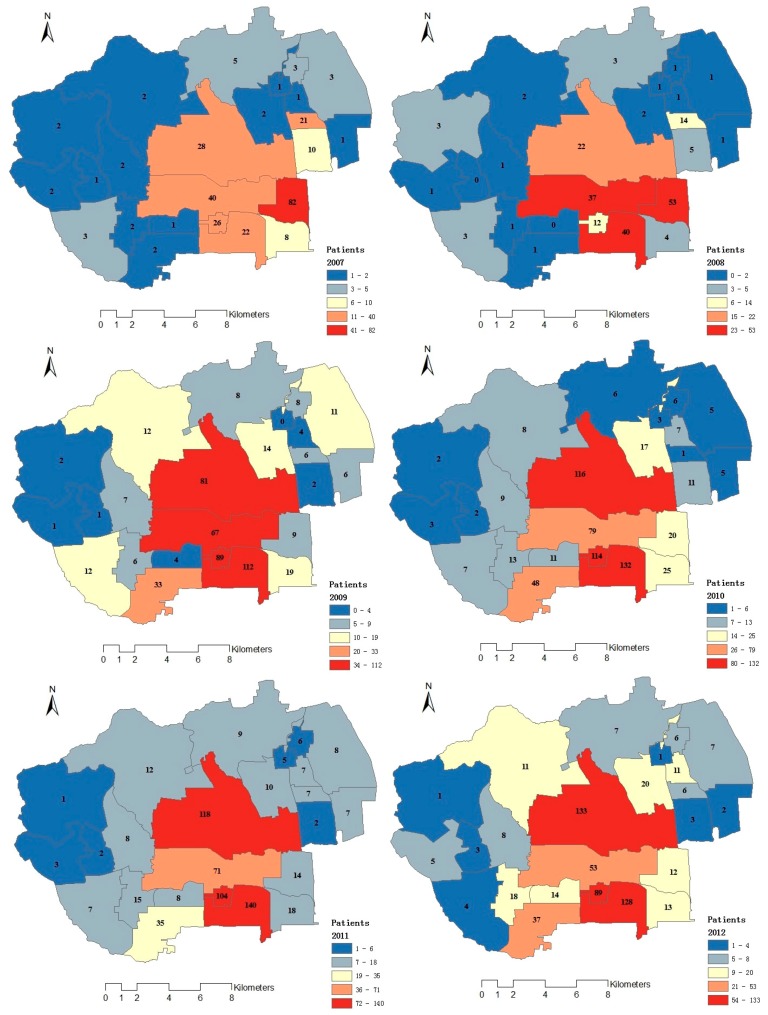
Spatial distribution of outpatients from 2007 to 2012.

The spatial distribution of outpatients’ locations was also attained from the information of outpatients’ addresses and zip codes. Results are shown in Figure 3. Overall, the spatial distribution of outpatients showed the following characteristics. Firstly, the spatial distribution of outpatients in different years varied considerably. Secondly, the spatial variation of outpatients was complex. Thirdly, due to the radiation scope of the hospitals, outpatients with respiratory system disease in this study were mainly concentrated in the southeastern regions (Balizhuang sub-district office, Ganjiakou sub-district office, Wanshoulu sub-district office, and Yongdinglu sub-district office) and east-central regions (Zizhuyuan sub-district office and Shuangyushu sub-district office) of the study area. Correspondingly, PM_1_ concentrations in these regions were higher than those in other regions. In turn, the numbers of outpatients in these sub-district offices heavily stricken by PM_1_ were higher than those of other sub-district offices. Finally, the incidence of outpatients during 2007~2012 showed an annually increasing trend.

### 3.3. Grey Correlation Analysis 

Grey correlation analysis was used to analyze the relationship between particulate matter number concentration and the number of outpatients in the study area. Considering the “week effect” of respiratory system diseases and the influence of meteorological factors, DOW (day-of-the-week) was introduced into the grey correlation model along with temperature, relative humidity, and wind speed as a comparative sequence. With the calendar effect and meteorological factors taken, the associations from small to large were obtained in Table 3. From Table 3, it is obvious that the impact degree of PM_0.3_ on the number of outpatients is the largest among the three sizes of particles, except in 2012. The influence degree of PM_0.3_ on the number of outpatients is the largest, which is 0.8213 (in the years 2009 and 2010) and the influence degree of PM_1.0_ is the smallest, which is 0.6949 in 2012. Based on the order of correlation degree, it is clear that there is an obvious regularity in the results of the grey correlation analysis during 2007~2012. Overall, the correlation degree order of fine particles was PM_0.3_ > PM_0.5_ > PM_1.0_. It turned out that smaller fine particles had more effect on the cases of respiratory system disease than the larger particles. From the grey correlation analysis, the correlation degree of all fine particle concentration to the number of outpatients is high. It means that fine particulate matter has a great impact on respiratory system disease. 

**Table 3:** Grey correlation degree and its range during 2007~2012.

Year	2007			2008			2009		
PM	PM_0.3_	PM_0.5_	PM_1.0_	PM_0.3_	PM_0.5_	PM_1.0_	PM_0.3_	PM_0.5_	PM_1.0_
Correlation degree	0.7771	0.772	0.7595	0.7467	0.7402	0.7425	0.8213	0.8211	0.8068
Correlation range	1	2	3	1	3	2	1	2	3
**Year**	**2010**			**2011**			**2012**		
PM	PM_0.3_	PM_0.5_	PM_1.0_	PM_0.3_	PM_0.5_	PM_1.0_	PM_0.3_	PM_0.5_	PM_1.0_
Correlation degree	0.8213	0.8099	0.7909	0.7944	0.7825	0.7624	0.702	0.7064	0.6949
Correlation range	1	2	3	1	2	3	2	1	3

## 4. Conclusions

The paper is an environmental health study that demonstrates how serious fine particle pollution in Beijing is and its possible correlation with hospital admissions for respiratory diseases. The results showed that spatial distributions of fine particles were diverse and there was a certain association between respiratory health and fine particles. The finding is consistent with the results of Professor Tang’s research in 2010 [13]. Outpatients with respiratory system diseases in this study were mainly distributed in the southeastern regions (Balizhuang sub-district office, Ganjiakou sub-district office, Wanshoulu sub-district office, and Yongdinglu sub-district office) and east-central regions (Zizhuyuan sub-district office and Shuangyushu sub-district office) of the study area. Correspondingly, PM_1_ concentrations in these regions were higher than those in other regions. Grey correlation analysis results showed that the correlation degree between all fine particle concentration and the number of outpatients is high, and the smaller fine particles had more obvious effects on respiratory system diseases than the larger particles. It is similar to results from previous research [14,30,31]. This research reveals the serious conditions of fine particle pollution and its environmental health effect in Beijing, China.
